# A study to examine the ageing behaviour of cold plasma-treated agricultural seeds

**DOI:** 10.1038/s41598-023-28811-w

**Published:** 2023-01-30

**Authors:** Naeem Ahmed, Kim S. Siow, M. F. Mohd Razip Wee, Anuttam Patra

**Affiliations:** 1 Institute of Microengineering and Nanoelectronics (IMEN), Universiti Kebangsaan Malaysia, UKM, 43600 Bangi, Selangor Malaysia; 2 Chemistry of Interfaces Group, Luleå University of Technology, 97187 Luleå, Sweden

**Keywords:** Plant sciences, Chemistry, Engineering

## Abstract

Cold plasma (low pressure) technology has been effectively used to boost the germination and growth of various crops in recent decades. The durability of these plasma-treated seeds is essential because of the need to store and distribute the seeds at different locations. However, these ageing effects are often not ascertained and reported because germination and related tests are carried out within a short time after the plasma-treatment. This research aims to fill that knowledge gap by subjecting three different types of seeds (and precursors): Bambara groundnuts (water), chilli (oxygen), and papaya (oxygen) to cold plasma-treatment. Common mechanisms found for these diverse seed types and treatment conditions were the physical and chemical changes induced by the physical etching and the cold plasma on the seeds and subsequent oxidation, which promoted germination and growth. The high glass transition temperature of the lignin-cellulose prevented any physical restructuring of the surfaces while maintaining the chemical changes to continue to promote the seeds germination and growth. These changes were monitored over 60 days of ageing using water contact angle (WCA), water uptake, electrical conductivity, field emission scanning electron microscopy (FE-SEM) and X-ray photoelectron spectroscopy (XPS). The vacuum effect was also investigated to separate its effect from cold plasma (low pressure). This finding offers a framework for determining how long agricultural seeds that have received plasma treatment can be used. Additionally, there is a need to transfer this research from the lab to the field. Once the impact of plasma treatment on seeds has been estimated, it will be simple to do so.

## Introduction

The increased food demand, caused by the world population growth, necessitates massive agricultural production. According to the Food and Agriculture Organisation, the global population will reach 10 billion by 2050. Therefore, it will be necessary to improve the quality of current food production even if the food supply does not increase^1^. Global food shortages are also exacerbated by climate change, rapid industrialisation, and urbanisation^2^. As cultivable land is limited, the most feasible way to meet these food demands is to increase crop yield through different crop tools^2^.

Since most food crops are grown from seeds, therefore some seed priming and scarification techniques are used to increase plant growth and yield. As consumer demand for farms to operate sustainably and economically drives the adoption of new agricultural approaches. Urban farming with hydroponics, precision farming, vertical farming, soil management, plant breeding or genetic engineering, microbial farming nanoparticles, herbs, biodegradable products, biological products, and organic matters are just a few examples^3–7^. As a result, there is a constant desire to find new ways to conserve resources while ensuring the healthy growth of the seed into a complete plant without harming the environment. Simultaneously, the damaging environmental effects of crop farming must be kept to a minimum. The techniques mentioned above are time-consuming, labour-intensive, and inconsistent. There is need for an environmentally approachable, scalable, and cost-effective methodology to overawe these challenges.

As a result, cold plasma has been proposed as a cost-effective and environmentally-friendly solution to these issues^7^. Plasma is an ionised gas, also known as the fourth state of matter. Plasma can be classified into hot and cold plasma. Cold plasma is further classified into low-pressure and atmospheric-pressure plasma. As part of this framework, cold plasma-treatments in agriculture are being extensively researched. Hundreds of research articles claiming improved crop germination and growth have been published^8–10^. However, as most of these studies were conducted immediately after plasma-treatment, further studies are needed to determine the duration of efficacy. Also, it is necessary to know whether or not plasma-treated seeds are effective in the later dates.

Plasma-treatment causes physical and chemical changes in the seed's surface and makes the surface hydrophilic, allowing easy transfer of nutrients essential for germination^10^. However, this hydrophilicity and oxidation are not permanent and can be reversed over time in a similar way to synthetic polymers due to their similar molecular structures. In plasma-modified synthetic polymers, the reorientation of the surface layer and surface oxidation have been proposed as explanations for hydrophobic recovery, also known as ageing^11–13^.

Agricultural seeds’ surfaces also undergo a similar ageing process; therefore, planting the plasma-treated stored seeds at later dates makes their rate of germination doubtful. Hence, it is essential to comprehend the ageing mechanism and the long-term effects of cold plasma-treatment. The current study aimed to investigate the effect of cold plasma over a period of 60 days, as well as the recovery of hydrophilization. The ageing of plasma-treated seeds for three different crops (Bambara, chilli, and papaya) was studied using WCA, water uptake, electrical conductivity, FE-SEM and XPS analysis. Optical emission spectroscopy (OES) was used to do plasma diagnostics. This is the first study to look into the ageing behaviour of cold plasma-treated agricultural seeds; this investigation will help both the researcher and the commercial planters of various crops.

## Results and discussions

### Water contact angle (WCA)

Firstly, the ageing behaviour was investigated based on the hydrophilicity of the substrate surfaces and their hydrophobic recovery over time. Surface hydrophilicity is reflected with a decrease in WCA. The current WCA study was carried out for a period of 60 days to investigate the effect of cold plasma-treatment on the seeds’ hydrophilicity and their ageing behaviour.

**Figure 1:**
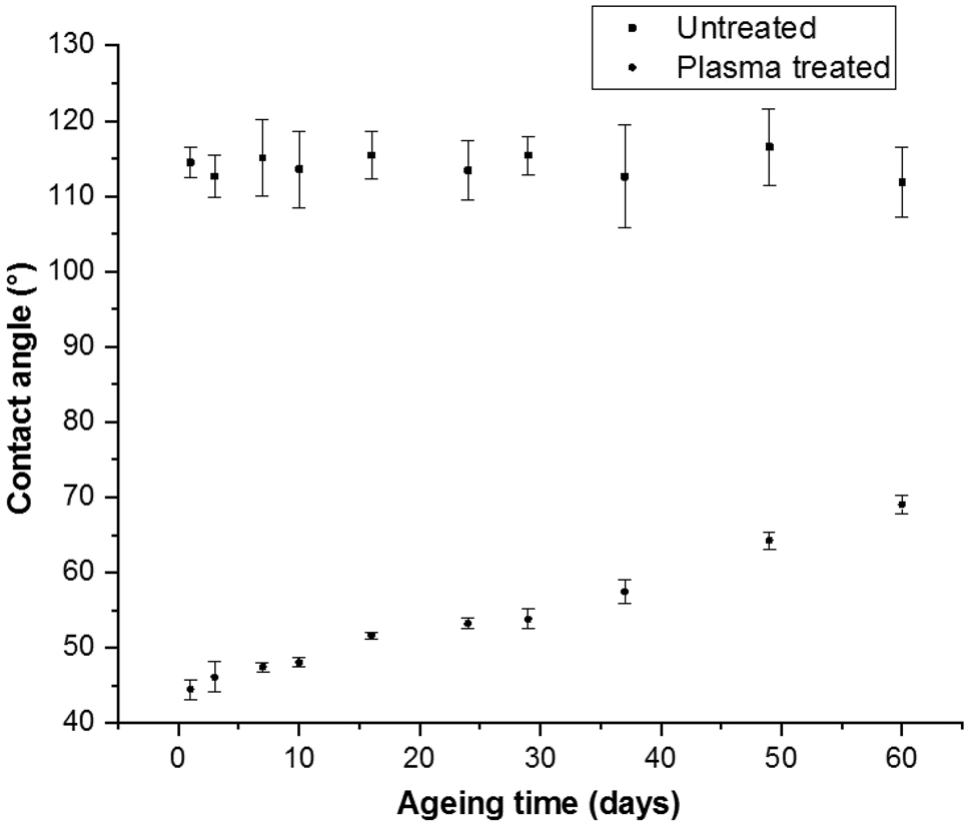
The WCA of untreated and water plasma-treated Bambara groundnuts for the ageing time of 60 days. The samples were treated for 10 s at a discharge power of 10 W. Experiment was performed in triplicates and error bars represent the standard deviation values*.*

**Figure 2:**
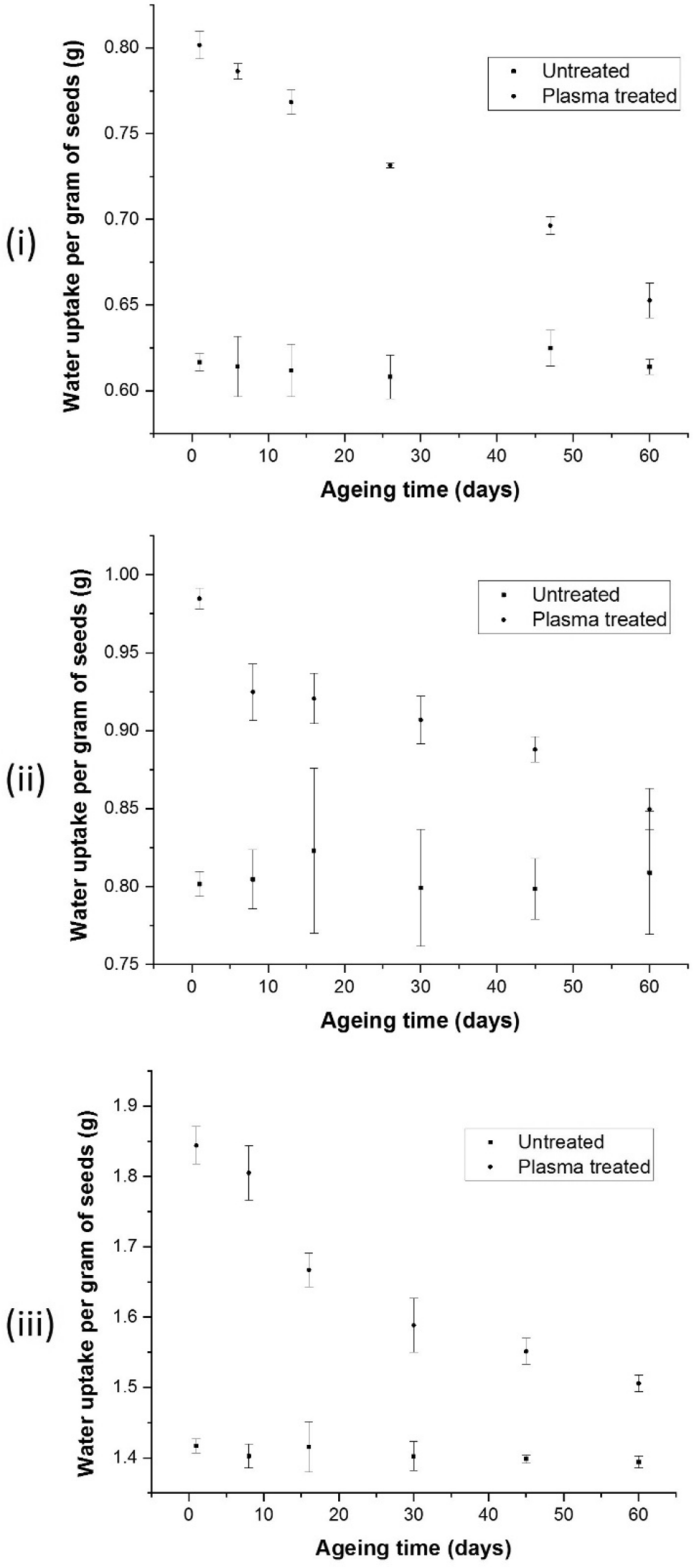
The difference in water uptake values for untreated and plasma-treated seeds of Bambara, chilli, and papaya is shown in sub-figures (**i**), (**ii**), and (**iii**), respectively. Water plasma was used to treat Bambara groundnuts, while oxygen plasma was used to treat chilli and papaya. Reading was recorded for 60 days to investigate the ageing behaviour of plasma-treated seeds. The experiment was repeated three times, and the error bars represent the standard deviation values. The water uptake results of Bambara, chilli, and papaya seeds treated with cold plasma were significant, with p values of 0.000, 0.001, and 0.004, respectively.

The WCA reduced from 114° to 44° for Bambara seeds, as shown in Fig. 1, and the recovery of WCA reached 69° after 60 days. A similar reduction in WCA was studied elsewhere, where the WCA reduced from 115° to 0° for the wheat seeds after plasma-treatment^8^. Our current findings were compared to those obtained from polymer surfaces with a large dataset for a better understanding of ageing phenomena, because crop seeds are essentially lignin and cellulose, composed of glucose and other small precursors. In the case of synthetic polymers, different degrees of hydrophobic recovery existed for different plasma polymers reported in the literature^13,14^. The difference between our and published results for synthetic polymers or seed crops (like wheat seeds) could be attributed to the different seeds having different inherent morphology and different plasma-treatment condition. Moreover, the chemical composition of seeds’ surface can vary a bit between different species, although basic chemistry of seed surface is more or less similar. The FE-SEM micrographs in Fig. 4 also suggest the plasma-etched seeds have improved hydrophilicity because their corrugated and porous surface absorbs the water more rapidly than those non-plasma-treated, illustrated in the water uptake section (Fig. 2). The seed surface contains of hilum and micropyle, which are important in the first stages of seed germination. A micropyle is a small pore near the hilum. It is through this pore that the germinating seed absorbs water and also where respiratory gases diffuse. These seed parts are encapsulated in a wax layer. The interaction of active plasma species with the surface of the seeds after plasma treatment removes the wax layer by oxidation and makes the hilum corrugated and micropyle porous. Further studies with the water uptake tests and FE-SEM, which will be discussed next, supported this suggestion. Residual hydrophilicity in the Bambara seeds ensures that the plasma-treated seeds can be used later after 60 days of storage for observation (Fig. 1). This WCA study was limited only to Bambara seeds, but not to chilli and papaya seeds. The non-uniformity and small size of the chilli and the hydrophilic nature of the papaya seeds prevented any repeatable and reliable measurement of WCA.

### Water uptake

Water uptake is a critical technique in germination and growth studies. An increase in water uptake values suggested that the nutrients are transferred to enhance the seeds’ germination and growth. As mentioned in the introduction part, the water uptake values of various crops significantly improved after cold plasma-treatment. This study demonstrated similar results with the water uptake values for cold plasma-treated seeds showing significantly higher values than those reported for the untreated seeds; Fig. 2i,ii,iii show the water uptake values of Bambara, chilli, and papaya seeds, respectively. Unlike the WCA studies, the water uptake values were recorded for 60 days for all three seeds.

Since the current study used the low-pressure plasma approach, the effect of vacuum on the water uptake needed to be investigated separately. Supplementary section Fig. S1a,b compare the water uptake values of the untreated with the vacuum-treated chilli and papaya seeds. The water uptake values of vacuum-treated seeds were associated with the moisture being pumped out from the seeds and then the rapid absorption of water during the water uptake test. As previously mentioned, after plasma treatment, the water uptake values significantly increased; however, after 60 days of ageing, the water uptake decreased, but the plasma-treated seeds' water uptake values were still higher at day 60 than those reported for untreated seeds. Furthermore, our results showed that vacuum also influences the water uptake value, but its effectiveness subsided by day 60, showing identical water uptake values for untreated and vacuum-treated seeds (Fig. S2). Thus, the plasma-treatment was mainly responsible for the increased water uptake for the plasma-treated seeds.

Plasma-treatment is useful to remove the wax layer from many seed surfaces to convert them to hydrophilic. The hydrophobic recovery may take part in the reduction of water uptake, but this recovery is insignificant until day 30. The water uptake results for plasma-treated seeds are significantly higher than untreated seeds on day 30. The increase and decrease in water uptake values were associated with physical and chemical changes in the seed's surface discussed in the following section.

### Electrical conductivity

**Figure 3:**
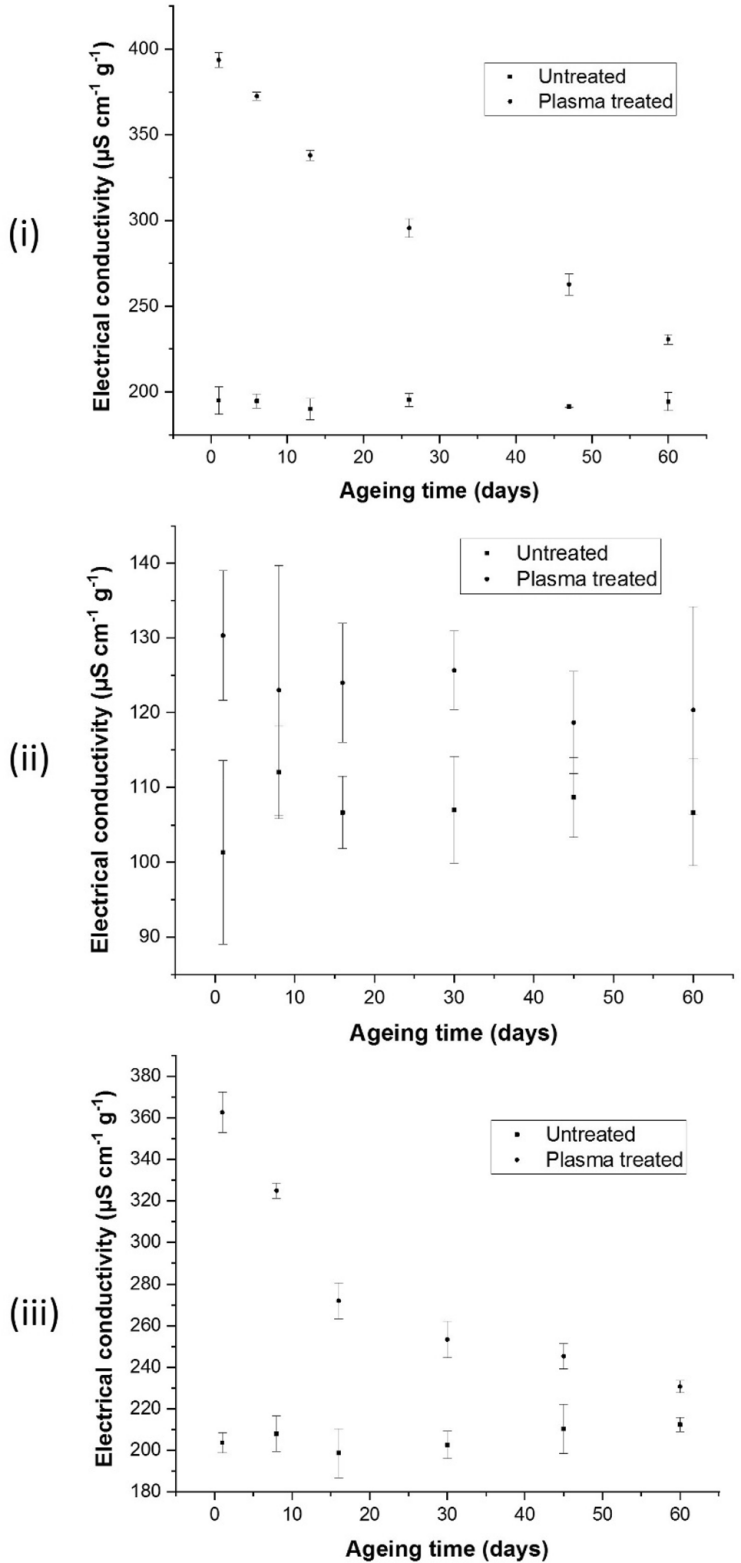
The difference in electrical conductivity values for untreated and plasma-treated seeds of Bambara, chilli, and papaya is shown in sub-figures (**i**), (**ii**), and (**iii**), respectively. Water plasma was used to treat Bambara groundnuts, while oxygen plasma was used to treat chilli and papaya. Reading was recorded for 60 days to investigate the ageing behaviour of plasma-treated seeds. The experiment was repeated three times, and the error bars represent the standard deviation values. The electrical conductivity results of Bambara, chilli, and papaya seeds treated with cold plasma were significant, with p values of 0.001, 0.000, and 0.002, respectively.

Excessive exposure to cold plasma weakens cell membranes, allowing intercellular molecules like sugars and electrolytes to escape out. These leached substances increase the electrical conductivity of water containing cold plasma-treated seeds, indicating the seed vigour. In this study, electrical conductivity values for cold plasma-treated seeds were significantly higher than those recorded for the untreated seeds. These electrical conductivity values for cold plasma-treated seeds had decreased with time but were still higher than untreated seeds after 60 days of air ageing. This reduction in electrical conductivity could be attributed to the decrease of the reactive species on the seed surface, such as radicals with a short life span post-plasma-treatment. The electrical conductivity values of Bambara, chilli and papaya seeds are represented in Fig. 3i,ii,iii, respectively. Similar to the water uptake test, the electrical conductivity of vacuum-treated seeds was also investigated to eliminate the influence of vacuum on the leaching process. As expected, the electrical conductivity values of vacuum-treated as well as untreated chilli and papaya seeds were nearly identical (Fig. S2).

In addition, the oxygen plasma produced ions that etched and oxidised the seed hilum and testa, as confirmed by the XPS measurements (Fig. 5). These oxidising and etching ions had a longer-lasting effect than the shorter-lived radicals that decayed over time. These ions were more likely to sustain the electrical conductivity effects reported in these studies. The lack of a vacuum influence on electrical conductivity (Fig. S2) further demonstrates that the vacuum only increases the water uptake values, but not the chemical composition.

An increase in electrical conductivity confirmed the leaching of nutrients, electrolytes, and salt contents from the seeds, indicating the seed vigour. However, water uptake and electrical conductivity are not always correlated directly to seeds germination and growth improvement. For example, the higher dosage of plasma-treatment increases the water uptake and electrical conductivity values by damage of the seed surface. This damage hurts the seeds’ germination and growth; higher electrical conductivity is also associated with more dead seeds, as reported elsewhere^15^. On the other hand, a moderate rise in electrical conductivity was associated with the positive interaction between water uptake and active plasma species introduced on the seed surface by cold plasma-treatment as described in our previous studies^10,16,17^. This moderate rise in electrical conductivity is useful for germination and growth; the leaching of nutrients and sugar contents was useful to provide the initial energy for germination^17^.

### FE-SEM

**Figure 4:**
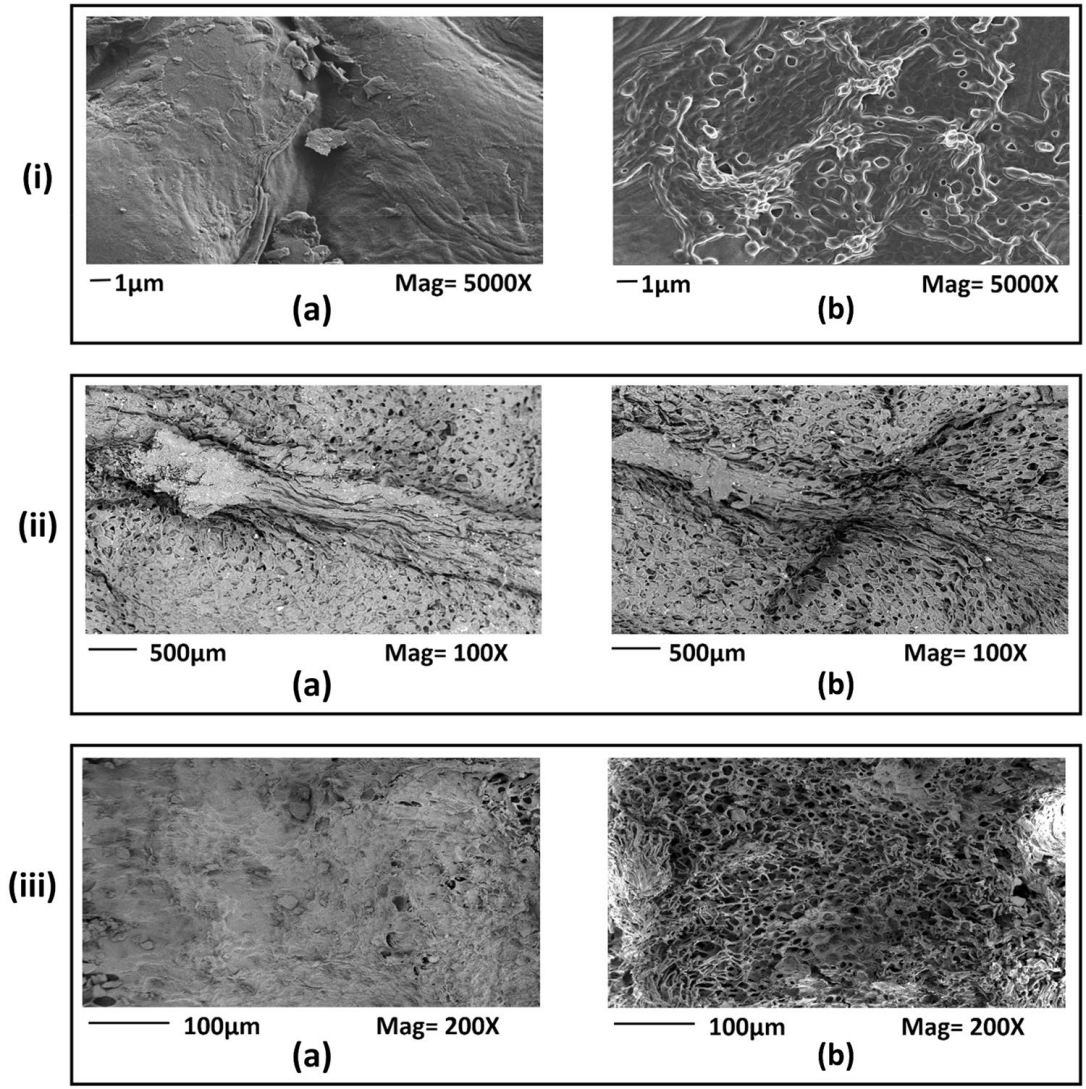
The difference between untreated and plasma-treated surfaces for Bambara, chilli and papaya was illustrated in sub-figures (**i**), (**ii**), and (**iii**), respectively. In sub-figures (**i**), (**ii**), and (**iii**) (**a**) represent the untreated seed surface, while (**b**) represent the surface of the plasma-treated seed. Bambara groundnuts were treated with water plasma, while the chilli and papaya were treated with oxygen plasma.

FE-SEM experiments were carried out immediately after plasma-treatment to investigate the physical changes in the surfaces of Bambara, chilli, and papaya seeds, and are shown in Fig. 4i–iii, respectively, which presents how cold plasma-treatment etches the morphology of seeds. The seed surfaces of cold plasma-treated Bambara, chilli, and papaya seeds become porous compared to the untreated seeds. Other researchers have reported similar surface changes as reported in the current investigation. For example, air plasma-treatment is used to modify the surface of wheat, beans, and lentil seeds in a similar way^8^. Their FE-SEM investigation corroborated the physical changes during the post-plasma-treatment, but their study was not extended for 60 days to account for ageing. Because plasma oxidation brought the surface chemical changes, ageing studies were performed in the current manuscript using XPS studies to investigate these oxidative changes. This FE-SEM study also supported the results for water uptake, WCA, and electrical conductivity.

### XPS

**Figure 5:**
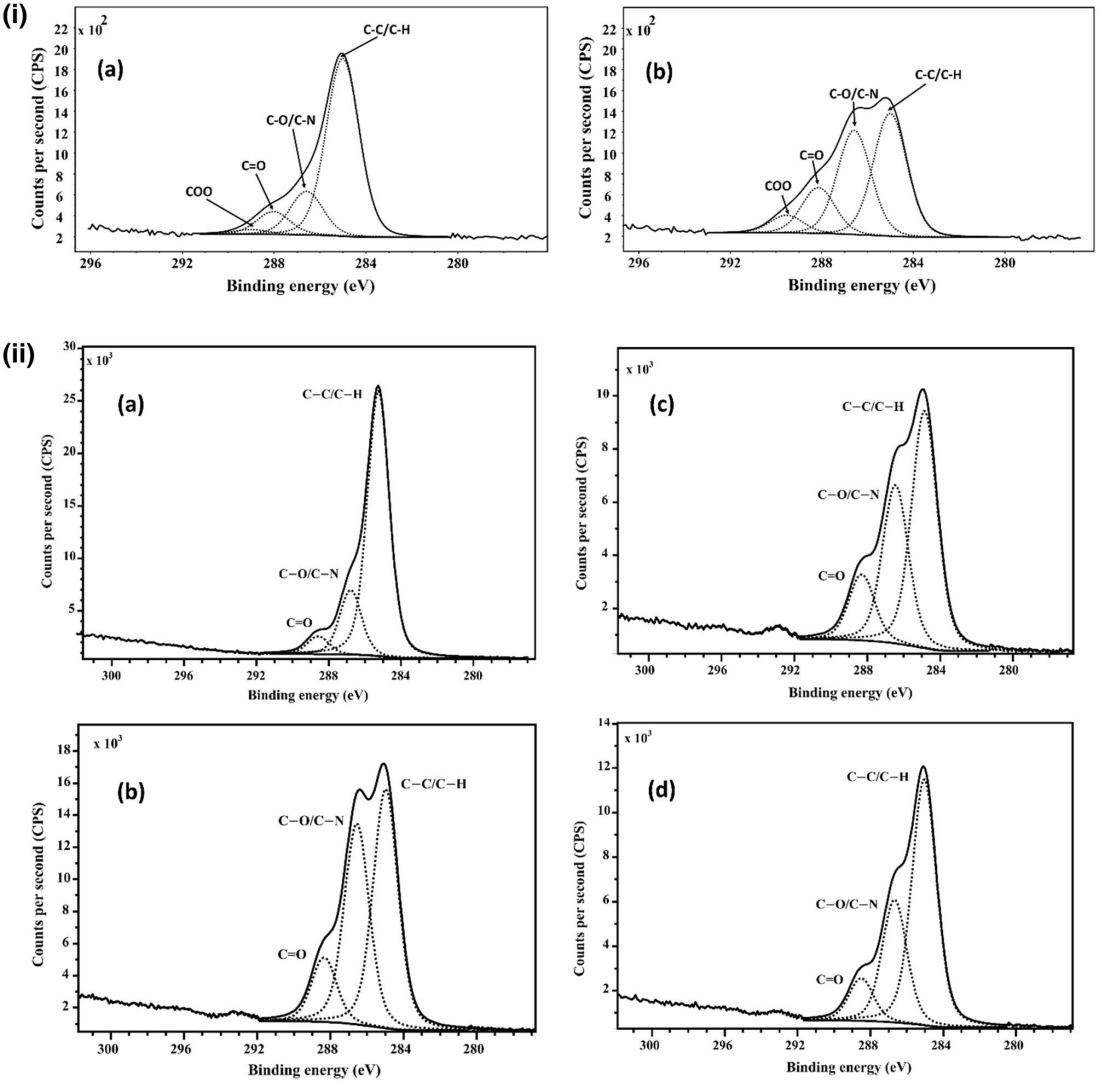

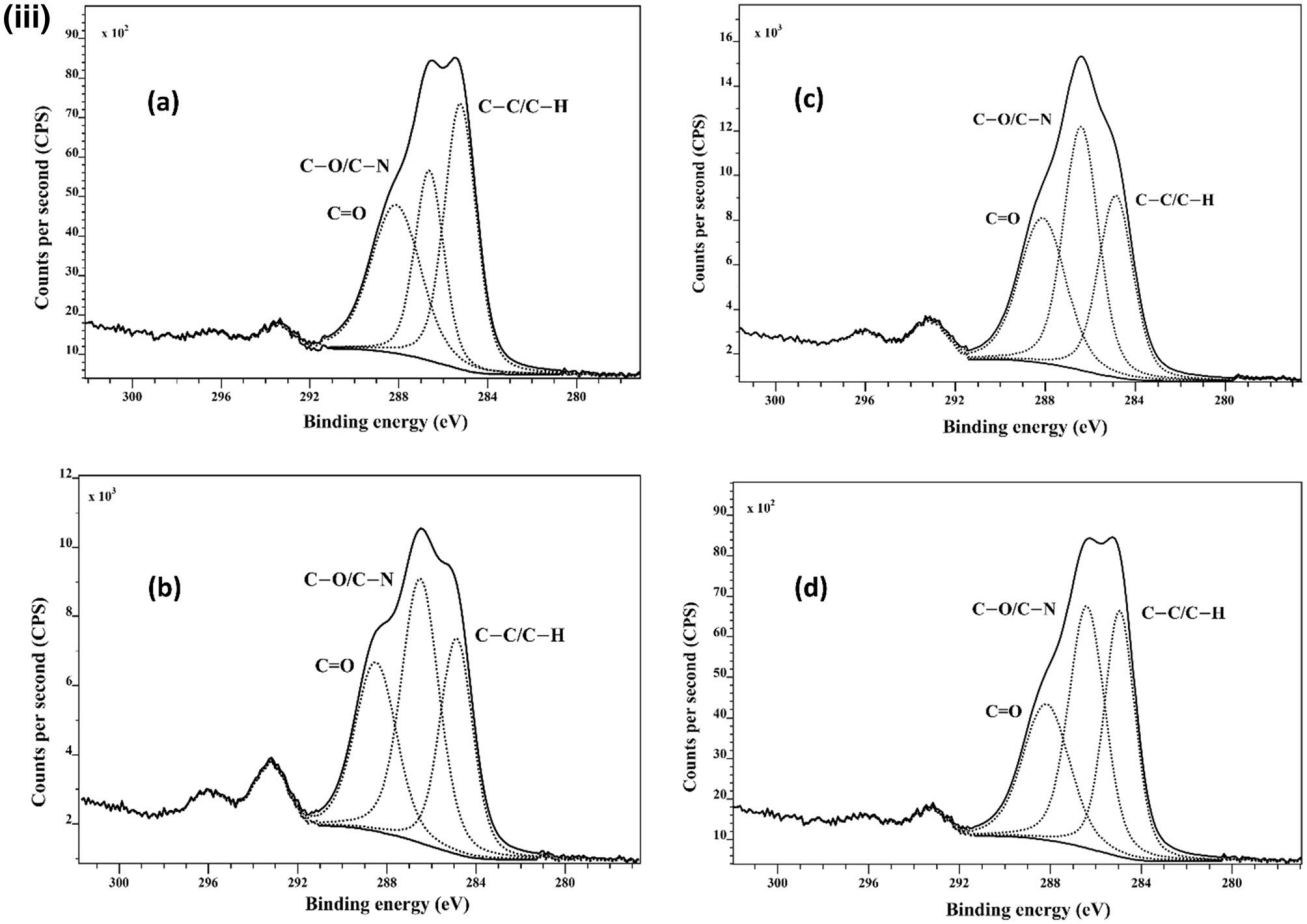
(**i**) Component fitting of high-resolution C1s spectra of (**a**) untreated and (**b**) water plasma-treated Bambara seeds. Bambara seeds were exposed to water plasma for 10 s at a discharge power of 10 W. (**ii**) Component fitting of high-resolution C1s spectra for untreated, oxygen plasma-treated chilli seeds on days 4, 21 and 51 is illustrated in sub-figures (**a**–**d**), respectively. Chilli seeds were exposed to oxygen plasma for 60 s at a discharge power of 80 W. To investigate the ageing behaviour, the XPS was carried out on the above-mentioned days. (**iii**) Component fitting of high-resolution C1s spectra for untreated, oxygen plasma-treated papaya seeds on days 4, 21 and 51 is illustrated in sub-figures (**a**–**d**), respectively. Papaya seeds were exposed to oxygen plasma for 40 s at a discharge power of 80 W. to investigate the ageing behaviour, the XPS was carried out on the above-mentioned days.

XPS was used to investigate the surface chemistry of Bambara, chilli, and papaya seeds using component-fitting of high-resolution C1s peaks. The chemical composition of cold plasma-treated seeds differs significantly from untreated seeds, based on the component-fitting results shown in Fig. 5. The XPS spectra of Bambara, chilli, and papaya seeds are shown in Fig. 5i,ii,iii respectively. The presence of several carbon bonds was revealed by deconvolutions of high-resolution C1s spectra of Bambara seeds (Fig. 5i): C–C/C–H (285 eV), C–O, C–N (286.5 eV), and C=O (288.3 eV)^10,18,19^. The experiment was repeated with chilli and papaya, and the effect was evaluated for a 51-day ageing period. Figure 5ii,iii reveal that after cold plasma-treatment, oxygen-related moieties rose, and remained more elevated in the aged seeds (21 and 51 days) than those reported for the untreated seeds. The surface chemical content of vacuum-treated seeds did not differ significantly from those reported for untreated seeds (Figs. S3, S4, and Table 1). As expected, this XPS result showed that the vacuum treatment only influenced the seed water uptake, as initially disclosed, not on surface chemistry or electrical conductivity reported earlier.

**Table 1:** The atomic percentage of the different elements present in Bambara, chilli and papaya seeds.

	Untreated	Vacuum-treated	Plasma-treated day 4	Plasma-treated day 21	Plasma-treated day 51
Chilli seeds using oxygen plasma					
C (%)	81.7	83.8	65.6	67.7	72.5
O (%)	16.9	15.1	30.2	29.0	24.4
N (%)	1.4	1.1	4.2	3.3	3.1
O/C (%)	0.2	0.2	0.5	0.4	0.3
N/C (%)	0.2	0.1	0.6	0.5	0.4
C–C or C–H (%)	62.3	61.9	34.8	34.8	43.4
C–O or C–N (%)	14.8	16.9	23.2	23.2	21.6
C=O (%)	4.2	5.0	9.7	9.7	7.5
Papaya seeds using oxygen plasma					
C (%)	63.3	61.8	45.1	52.6	61.8
O (%)	29.4	29.7	43.9	40.8	33.6
N (%)	6.8	7.0	4.8	3.6	4.0
F (%)	0.0	0.0	0.9	0.0	0.0
Mg (%)	0.0	0.0	1.5	0.5	0.6
Si (%)	0.5	1.5	1.9	1.2	0.0
S (%)	0.0	0.0	0.6	0.0	0.0
Ca (%)	0.0	0.0	1.3	0.5	0.0
O/C (%)	0.4	0.4	1.0	0.8	0.5
N/C (%)	0.1	0.1	0.1	0.1	0.1
C–C or C–H (%)	25.9	27.4	13.2	14.1	14.1
C–O or C–N (%)	19.6	16.7	21.3	22.9	20.1
C=O (%)	17.8	17.7	10.6	15.2	13.9

	C (%)	O (%)	N (%)	O/C	N/C
Bambara seeds using H_2_O plasma					
Untreated	81.0	15.0	4.0	0.2	0.05
Plasma-treated	67.8	27.2	5.0	0.4	0.07

The compositions of the Bambara, chilli and papaya seeds are described in detail in Table 1. In all three crops, the O/C ratio of plasma-treated seeds increased compared to those reported for the untreated and vacuum-treated seeds. Compared to untreated seeds, these O/C ratios decreases with time, although they remained elevated till day 51. In addition to carbon, oxygen, and nitrogen, other low-concentration elements reported were calcium, silicon, magnesium, sulphur, aluminium, and fluorine (Table 1). These elemental concentrations totalled approximately 2%, with no apparent relationship to the plasma-treatment; the presence of these elemental concentrations are associated with surface impurities.

Another possibility for these microelements is attributed to removal of organic components by reactive oxygen species (ROS), leaving inorganic compounds on the surface. The higher electrical conductivity also supports this supposition because inorganic substances might dissolve in water and increase conductivity. These microelements are also found elsewhere during XPS studies and it was supposed that due to plasma treatment the segregation of these microelements from the internal part of seeds occur which is also contributing to germination improvement^20,21^. In a recent study, the presence of microelements was also attributed to the removal of the top lipid layer which uncovered the microelements below^22^.

The oxygen-related moieties are significantly enhanced after plasma-treatment, as indicated in Table 1 and Fig. 5. For both oxygen and water plasma-treatment, the O/C ratio increased (from 0.2 to 0.4 for Bambara, 0.4 to 1.0 for papaya and 0.2 to 0.5 for chilli), whereas the N/C ratio increased for Bambara and chilli seeds (0.4–0.5 and 0.2–0.6) but the N/C ratios decreased for papaya seeds (0.107–0.106). However, this reduction was also reported elsewhere, when pepper and melon seeds were treated with a similar plasma source, but they differ in nitrogen concentration on the seeds^23^. These findings suggested that the precursors used in the plasma-treatment impacted oxygen and nitrogen moieties found on the surfaces.

Due to their similarities with the seed surfaces, made up of a "cellulose-lignin" structure with cross-linked lignols and polysaccharides, we proposed a possible mechanism for this ageing phenomenon similar to those reported in synthetic polymers. The ageing of these polymeric structures is governed by two main mechanisms: post-plasma oxidation and surface adaptation. The initial increase in O/C ratios was caused by radicals interacting with in-diffusing oxygen from the environment. The interfacial chemistry influenced the surface adaption, and the plasma-treated seed surface uniquely interacted with its surroundings. Mobile and immobile polar groups on the polymeric substrate cause surface reorientation. These factors are classed as internal and external ageing factors. Internal factors include interfacial enthalpy, entropy and cross-linking density, while external factors contain contaminant adsorption, oxidation and temperature^12^. Internal factors influence polymer chain mobility, whereas external factors pertain to the environment's effect on ageing behaviour.

Although this surface reorientation is commonly observed in the synthetic polymer, this mechanism is unlikely to be operational for hard testa coats, which tend to have a relatively high glass transition temperature of more than 0 °C^24^. Hence, seed type and plasma modification type play a significant impact on the internal and exterior components of these ageing processes. Furthermore, the ageing of these materials frequently involved more than just oxidative chemical changes; over time, the physical process of surface remodelling also occurred, resulting in measurable changes in WCAs. However, this study concluded that the influence of germination and growth-promoting factors^9,23^ are present during 60 days of ageing, but those are most significant within the first 30 days.

### OES

**Figure 6:**
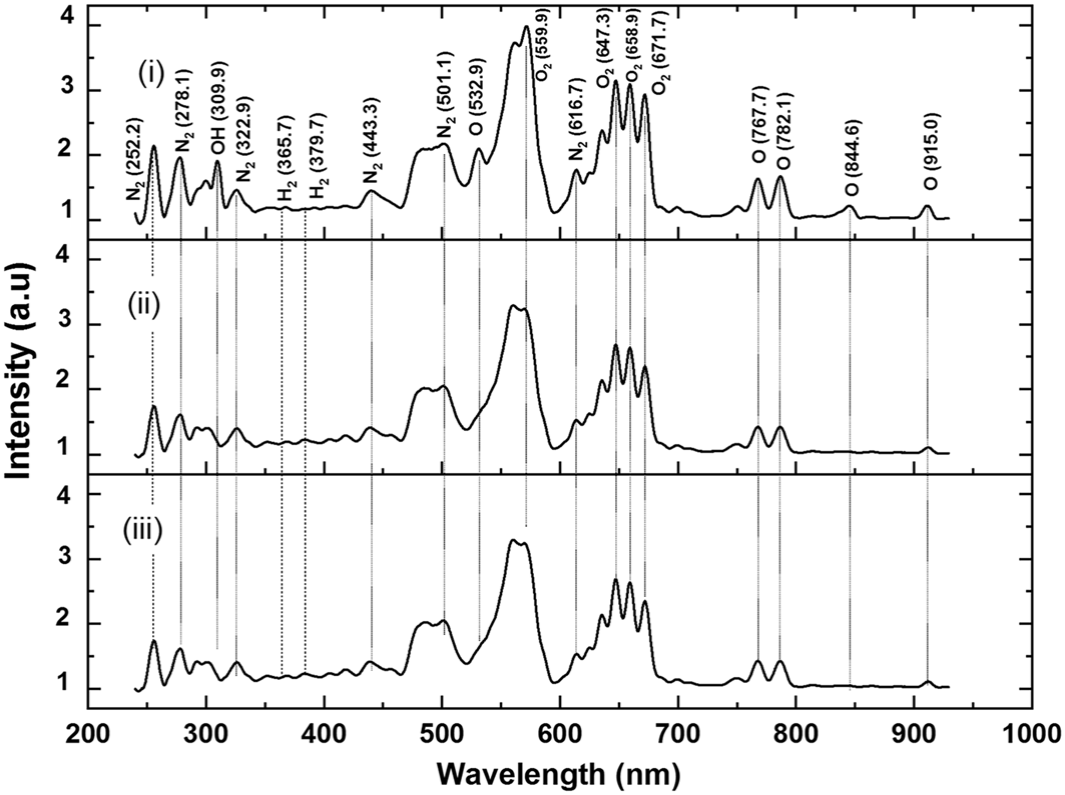
Sub-figure (**i**) represents the spectra obtained during Bambara seeds treatment using water plasma at a discharge power of 10 W for an exposure time of 10 s. Sub-figure (**ii**) represents the spectra obtained during chilli seeds treatment using oxygen plasma at a discharge power of 80 W for an exposure time of 60 s. Sub-figure (**iii**) represents the spectra obtained during papaya seeds treatment using oxygen plasma at a discharge power of 80 W for an exposure time of 40 s.

Figure 6 depicts the optical emission spectra obtained in the 200–1000 nm spectral region. Oxygen, nitrogen, hydrogen, and OH peaks dominated these spectra. The spectra for Bambara, chilli and papaya seeds are represented in sub-Figs. 6i,ii,iii respectively. Water plasma was used for the Bambara seeds, whereas oxygen plasma was used for the chilli and papaya seeds for varying exposure times. The species generated during plasma-treatment used in the surface modification; nitrogen species are produced between 294 to 300 nm. From 350 to 380 nm, the spectra also showed the presence of hydrogen lines and CO bands. The oxygen and nitrogen species overlapped the band from 390 to 950 nm^25–27^. In Fig. 6i, OH group is present at 309 nm for the water plasma-treated Bambara groundnut. In contrast, this OH group is absent during oxygen plasma-treated chilli and papaya seeds, shown in Fig. 6ii,iii, respectively.

The spectra for oxygen plasma are shown in sub-Fig. 6ii,iii, and they are identical, indicating that the variation in exposure duration did not affect the optical emission spectra. In the oxygen spectra, oxygen was dominant for surface etching because of its etching nature. Water plasma has 25 times higher etching ability than other precursors^17^, etching Bambara seeds with a 10 W discharge power and a 10-s exposure time was sufficient to affect the germination process. The appearance of nitrogen and hydrogen species in the oxygen spectra was attributed to air remaining in the chamber during plasma-treatment.

### Statistical analysis

Electrical conductivity and water uptake studies were both subjected to statistical analysis (shown in Table S1). The water uptake results of Bambara, chilli, and papaya seeds treated with cold plasma were significant, with p values of 0.000, 0.001, and 0.004, respectively. Similarly, the electrical conductivity results of Bambara, chilli, and papaya seeds treated with cold plasma were significant, with p values of 0.001, 0.000, and 0.002, respectively. Cold plasma-treated chilli and papaya seeds had significantly higher electrical conductivity than vacuum-treated seeds. Water uptake results of the plasma-treated seeds were insignificant compared to vacuum-treated seeds because vacuum also induced “hygroscopic” properties to encourage water uptake in the seeds. The results of vacuum-treated seeds in terms of water uptake and electrical conductivity were insignificant (Table S1) compared to untreated seeds, except papaya remained at the transition stage.

## Material and methods

### The source of seeds

In the current study, three crop species were used to examine their ageing behaviour after cold plasma-treatment. Crops For Future, Malaysia provided the Bambara seeds (*Vigna subterranean*), while the Malaysian Agricultural Research and Development Institute (MARDI) provided the chilli (semerah) and papaya (Eksotika). Healthy crop samples with no signs of infection were used in the current study. The seeds verification letter is available in supplementary data, and all methods included in the study are in accordance with international guidelines.

### Cold plasma-treatment

Cold plasma-treatment was carried out similar to our previous studies^10,28^. The parameters selected for germination and growth improvements were also utilised for the ageing studies. Every crop species responds differently to plasma-treatment, and hence the exposure time as well as discharge power also differ for every individual species. Plasma-treatments were carried out in a custom-built reactor with an upper U-shaped copper electrode and a bottom circular copper electrode having a 10 cm diameter at a distance of 15 cm from the other electrode. A radio-frequency generator (RF-3-XIII) was used to generate the plasma operating at 13.56 MHz. A vacuum pump (*Edwards 8*) was used to pump down the pressure until 6.6 Pa. The Edwards RV8 rotary vacuum pump features a peak pumping speed of 6 cfm at 60 Hz and an ultimate pressure of 1.5 × 10^–3^ Torr. (Schematic diagrams for the setup used in the current study are presented Figs. S5^17^ and S6). The pressure monitoring was carried out using a CVM211 stinger vacuum gauge. The Bambara seeds were treated using water as a precursor. The water was fed into the chamber through a needle valve. The pressure reached 40 Pa, which further rose to 46 Pa during plasma ignition. 20 Bambara seeds were treated simultaneously for a 10 s time duration at a discharge power of 10 W. The pressure was also reduced to 6.6 Pa for papaya and chilli seeds, after which pure oxygen (99.9%) was fed through a needle valve till the pressure increased to 35 Pa. In the case of chilli and papaya, the pressure rose to 40 Pa during plasma ignition at a discharge power of 80 W. Chilli seeds were exposed for 60 s, whereas papaya seeds were exposed for 40 s. Due to size differences, 50 papaya seeds and 100 chilli seeds were treated separately during plasma-treatment.

### Vacuum treatment

In this current study, chilli and papaya seeds were treated to the same vacuum level (6.6 Pa) similar during plasma treatment. The vacuum treatment contains the same number of seeds as the respective plasma-treatment. Since the seeds were vacuumed before and during low-pressure plasma-treatment, the effect of vacuum during low-pressure plasma-treatment was investigated separately to clarify any deterministic effect.

### WCA

The WCA study was carried out according to established protocol^17^. The apparent WCA was measured using a macro-lens setup with a high-resolution camera and utilizing a deionised (DI) water droplet of ~ 5 μl volume, with five replicates. A software ImageJ (ver. 1.52a) was used to measure the WCA from their respective images. On the same day, under the same treatment conditions, about 100 seeds were treated. The WCA was measured using 5 seeds that were randomly chosen, and the remaining seeds were stored at 5 °C for use on subsequent days. The possibility of error prevents the seeds from being used again; otherwise, incorrect WCA values might have been obtained as a result of water droplets absorbed by the seeds, which can alter the moisture content percentage and impact the contact angle values. To study ageing behaviour, WCA changes were recorded for up to 60 days. Because of the irregular and small size of chilli and the hydrophilic nature of papaya, the WCA study was limited to Bambara seeds only.

### Water uptake

Water uptake measurements were carried out using an earlier described method with modifications^10,16^. Because of the considerable variation in size, each species has a different sample size. For chilli, papaya, and Bambara seeds, sample sizes of 0.2 g, 0.3 g, and 5 g were chosen and weighed on an electronic balance to a precision of 0.1 mg. Plasma-treated and untreated samples were separated into six groups. The measurements for chilli and papaya seeds were carried out on days 1, 8, 16, 30, 45, and 60, whereas the measurements for Bambara seeds were recorded on days 1, 6, 13, 26, 47, and 60. Chilli and papaya seeds were soaked in 10 ml DI water for 24 h, whereas Bambara seeds were immersed in 40 ml DI water for 24 h. After that electrical conductivity meter (model CTR-007, manufactured by Fuzhou Centre was used to measure the electrical conductivity of water containing chilli, papaya and Bambara seeds. The electrical conductivity was used to determine how much ions (electrolytes) leak from the protoplast space or seed apoplast. The remaining samples were stored at 5 °C in Petri dishes for use on subsequent days (discussed above) to investigate ageing.

### FE-SEM

The surface morphology studies of chilli and papaya seeds were carried out using FE-SEM (Carl Zeiss Supra 55VP), while for Bambara groundnuts FE-SEM (Hitachi SU8230) was used. The operating voltage for chilli and Bambara groundnut was 10 kV, while 3 kV was used for papaya. All samples were gold-coated before imaging.

### XPS

The surface chemical properties were investigated using an XPS system, namely a ULVAC-PHI Quantera II equipped with an Al K source with an energy of 1486.6 eV and a working voltage of 15 kV. Narrow and wide scan spectra were measured at pass energy of 112 eV and 280 eV with a resolution of 0.1 and 1 eV, respectively^10^. The CasaXPS^®^ software (ver. 2.3.22PR1.0) was used to component-fit high-resolution spectra of the C1s regions. The binding energy (BE) of the "neutral" C peak (i.e., C–C and C–H components) was assigned to 285.0 eV during component-fitting.

### OES

OES study was carried out using the method previously described^10,17^. Ocean Optics USB2000 was used to identify light radiations emitted during the glow of ionised gas and then analysed to evaluate the plasma species formed during plasma-treatment. An optical cable (727-733-2447) was placed 5 cm away from the plasma chamber for oxygen plasma and 3 cm away from the chamber for water plasma to record the emitted radiation in the dark. Ocean View software (version 2.0.7) was used together with USB2000 to record the spectra.

### Statistical analysis

Water uptake and electrical conductivity results were statistically analysed using Minitab 17. Means were compared using one-way ANOVA based on the Tukey–Kramer comparisons test and considered significantly different when p < 0.05.

## Conclusion

Cold plasma-treatment significantly reduced the WCA, while enhanced the water uptake, and electrical conductivity of the plasma-treated seeds. Increase in water uptake was also attributed to the effect of vacuum because the seeds were simultaneously exposed to vacuum during low-pressure plasma-treatment. The physical and chemical changes on the seeds acted simultaneously to facilitate the seed germination and growth, as investigated in our previous studies^10,16^. However, ageing deteriorated these enhanced properties amongst the plasma-treated seeds, which were reflected by a decrease in the measurement values of the different tests explained in methodology section for 60 days, albeit still higher than the untreated values. However, the water uptake, electrical conductivity, and XPS studies showed that the plasma-treated values are significantly higher than the untreated values till day 30, while the WCA values are lower than the untreated values for the same period, indicating that the seeds can be used for an extended period after plasma-treatment. The plasma-treatment effect was maintained because of the enduring physical–chemical changes on the seeds despite some recovery in the plasma ions effects.

## Supplementary Information

Supplementary Information 1.

Supplementary Information 2.

## Data Availability

The datasets generated and/or analysed during the current study are available in the attached supplementary raw file. The raw data file is available in the ‘Related files’ section. The attached folder contains the raw files for XPS, OES, water uptake, WCA and electrical conductivity. The text file also included a description of the XPS samples. Because we are using the original images for FESEM, we are not attaching the FESEM images.
