# Supplementary material for: A study to examine the ageing behaviour of cold plasma-treated agricultural seeds
Source: Sci Rep. 2023 Jan 30;13:1675. doi: 10.1038/s41598-023-28811-w (PMC9886913; doi:10.1038/s41598-023-28811-w)
Supplement: Supplementary file 1 — Supplementary Information 1. [file 41598_2023_28811_MOESM1_ESM.pdf]

KEMENTERIAN PERTANIAN DAN INDUSTRI MAKANAN

INSTITUT PENYELIDIKAN DAN KEMAJUAN PERTANIAN MALAYSIA
Malaysian Agricultural Research and Development Institute
Pusat Pengkomersialan Teknologi & Bisnes
Ibu Pejabat MARDI
Persiaran MARDI-UPM
43400 Serdang, Selangor Darul Ehsan
MALAYSIA

Tel: 03-8953 6900
Laman Web: www.mardi.gov.my
E-mel : idah@mardi.gov.my

Reference No. : MDI/CB/PA/1-14
Date : 10 January 2022

Ch Naeem Ahmed
IMEN, Universiti Kebangsaan Malaysia

SEED SAMPLE VERIFICATION

The following is a seed verification report for the seed samples requested for research purposes.

No of sample	Seed	Variety/Batch	Seed Variety Verification
1	Papaya	Eksotika/ E 181126	Verified
2	Papaya	Eksotika/ E 81219	Verified
3	Papaya	Eksotika/ E 190226	Verified
4	Papaya	Eksotika/ E 190120	Verified
5	Papaya	Eksotika/ E 190414	Verified
6	Chilli	Semerah/10/16/JK/B3 14032017	Verified
7	Chilli	Semerah/10/16/JK/B3 10032017	Verified
8	Chilli	Semerah/10/16/JK/B3 29112016	Verified

Thank you very much.

“PENERAJU INOVASI AGROTEKNOLOGI”

“WAWASAN KEMAKMURAN BERSAMA 2030”

“BERKHIDMAT UNTUK NEGARA”

Saya yang menjalankan amanah,

…………………………………………

(PN. ZAITIALIA BINTI MOHLISUN)
Pegawai Penyelidik
Program Pengeluaran Biji Benih dan Bahan Tanaman
Pusat Pengkomersialan Teknologi & Bisnes
