# Supplementary material for: A study to examine the ageing behaviour of cold plasma-treated agricultural seeds
Source: Sci Rep. 2023 Jan 30;13:1675. doi: 10.1038/s41598-023-28811-w (PMC9886913; doi:10.1038/s41598-023-28811-w)
Supplement: Supplementary file 2 — Supplementary Information 2. [file 41598_2023_28811_MOESM2_ESM.docx]

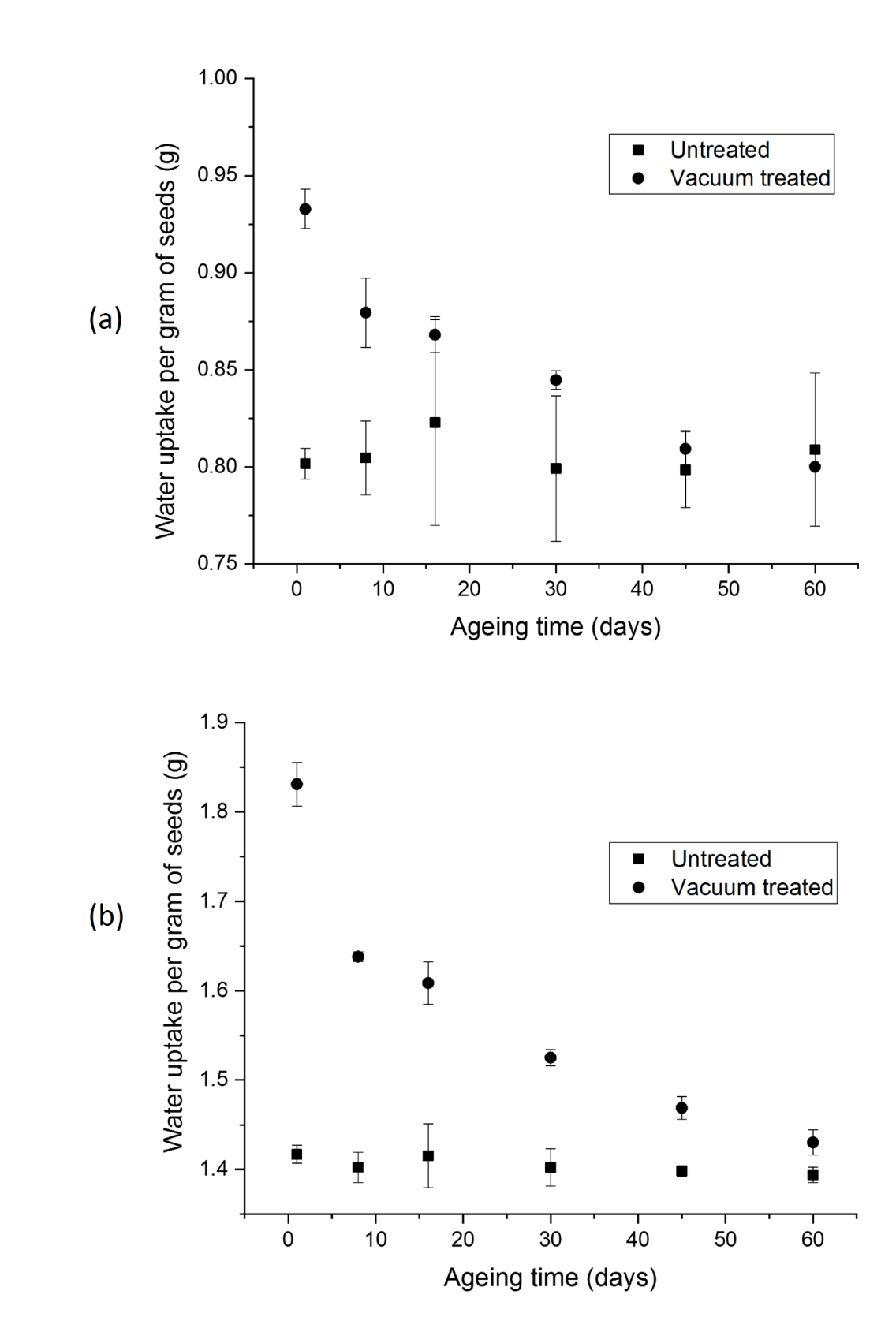


**Figure S1**. Comparison of the water uptake values for untreated and vacuum treated seeds. Sub-Figures (a) and (b) illustrate the water uptake values of chilli and papaya seeds respectively, for the ageing time of 60 days.


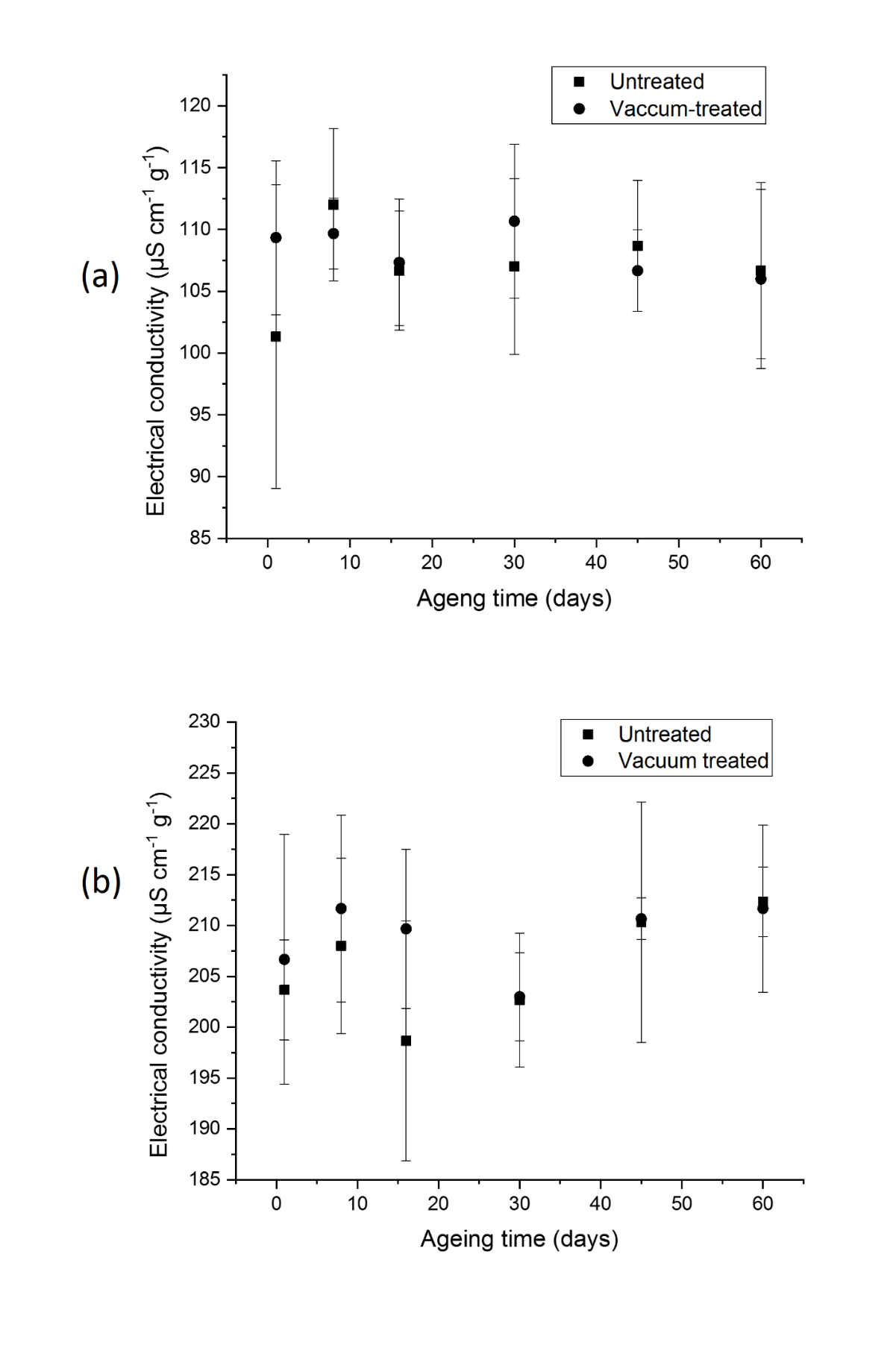


**Figure S2.** Comparison of the electrical conductivity values for untreated and vacuum treated seeds. Sub-Figures (a) and (b) illustrate the electrical conductivity values of chili and papaya seeds respectively, for the ageing time of 60 days.


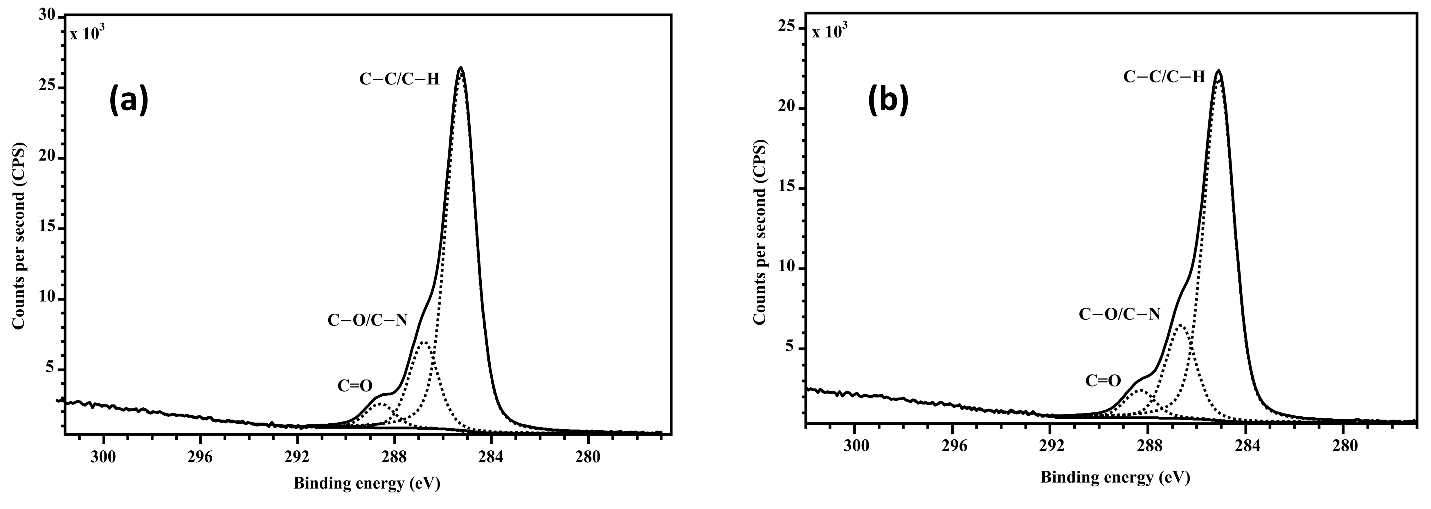


**Figure S3.** Component fitting of high-resolution C1s spectra of (a) untreated and (b) vacuum-treated chilli seeds.


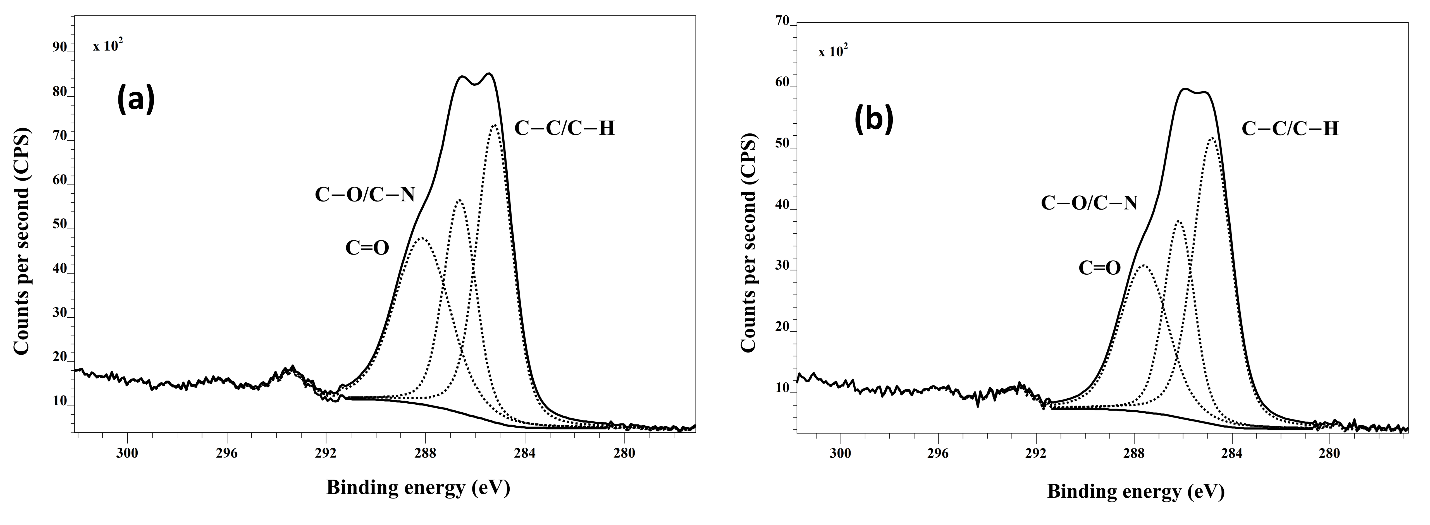


**Figure S4.** Component fitting of high-resolution C1s spectra of (a) untreated and (b) vacuum-treated papaya seeds.


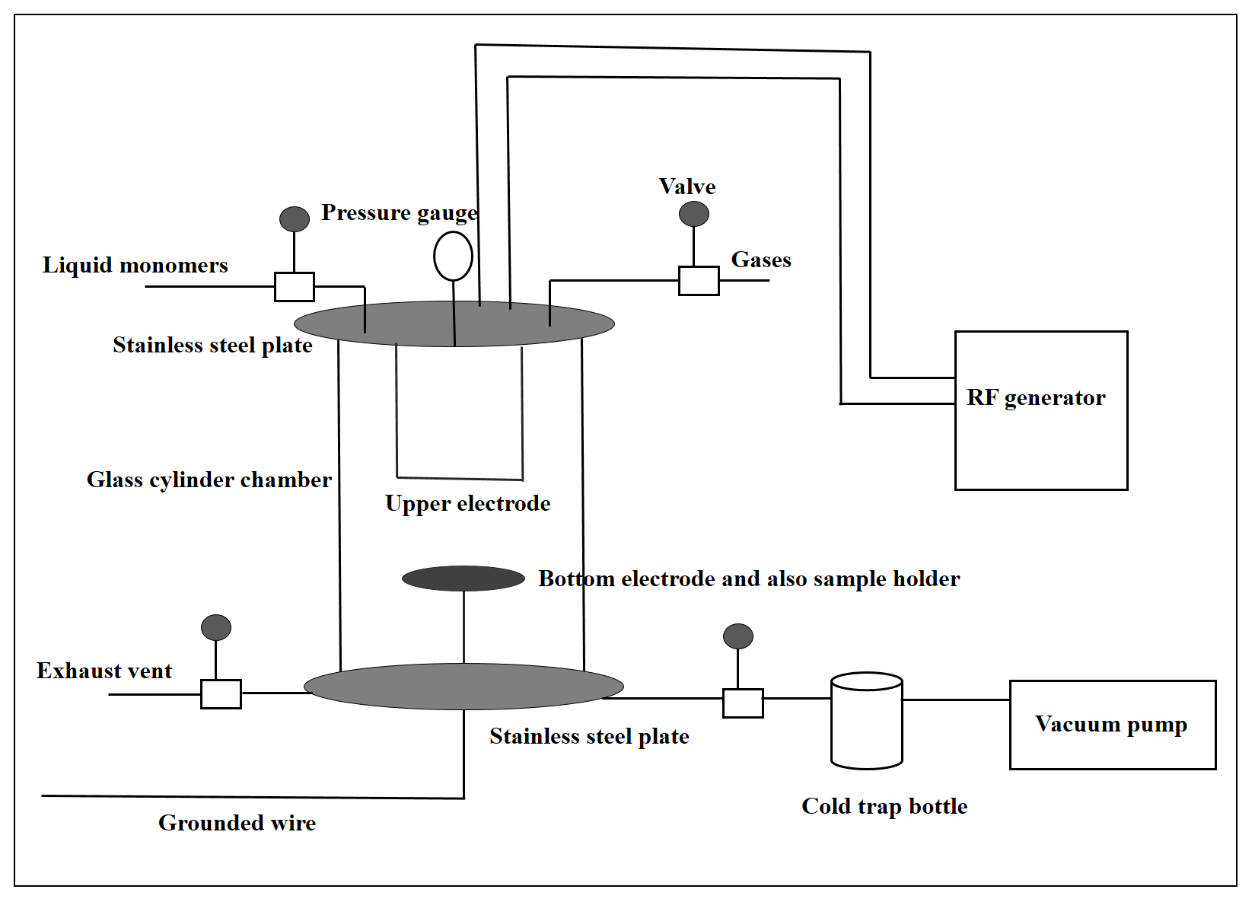


**Figure S5.** Schematic diagram for the low-pressure plasma setup


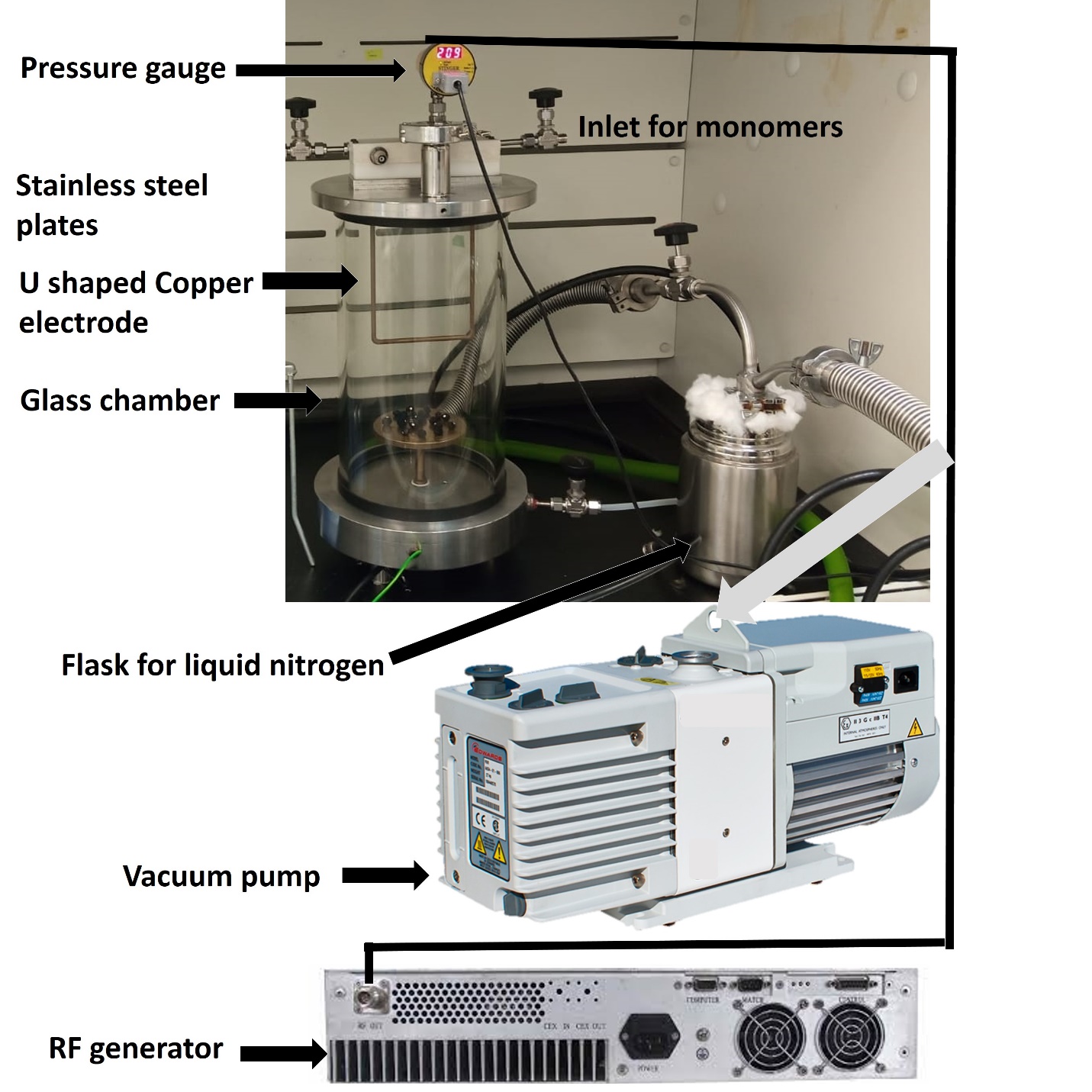


**Figure S6.** Low-pressure plasma setup used in the current study

**Table S1**. Statistical analysis results for water uptake and electrical conductivity

| ***p-* values for water uptake** | | | |
| --- | --- | --- | --- |
|  | Plasma-treated *vs*. Untreated | Plasma-treated *vs*. Vacuum-treated | Vacuum-treated *vs*. Untreated |
| Bambara | 0.000 | ---- | ---- |
| Chilli | 0.001 | 0.056 | 0.099 |
| Papaya | 0.004 | 0.501 | 0.043 |
| ***p-* values for electrical conductivity** | | | |
|  | Plasma-treated *vs*. Untreated | Plasma-treated *vs*. Vacuum-treated | Vacuum-treated *vs*. Untreated |
| Bambara | 0.001 | ---- | ---- |
| Chilli | 0.000 | 0.000 | 0.799 |
| Papaya | 0.002 | 0.002 | 0.984 |
